# C1QTNF6 regulates cell proliferation and apoptosis of NSCLC *in vitro* and *in vivo*

**DOI:** 10.1042/BSR20201541

**Published:** 2021-01-12

**Authors:** Wei Zhang, Ganzhu Feng

**Affiliations:** 1 Department of Respiratory Medicine, The Fourth Affiliated Hospital of Nanjing Medical University, Nanjing, Jiangsu, China; 2 Department of Respiratory Medicine, The Second Affiliated Hospital of Nanjing Medical University, Nanjing, Jiangsu, China

**Keywords:** apoptosis, C1QTNF6, NSCLC, proliferation

## Abstract

**Objectives:** Lung cancer has been reported as the leading cause of cancer-associated deaths in humans, and its incidence continues to increase in the world. A growing number of studies have shown that dysregulated genes are associated with the occurrence and poor prognosis of a variety of tumors, including non-small cell lung cancer (NSCLC). C1q/tumor necrosis factor-related protein 6 (C1QTNF6), a member of the C1q/tumor necrosis factor-related protein (CTRP) family, has been revealed to play a role in carcinogenesis and cancer progression. Nevertheless, the effects and mechanisms of C1QTNF6 in NSCLC remain unrevealed. **Materials and methods:** MTT (3-(4,5)-dimethylthiahiazo(-z-y1)-3,5-di-phenytetrazoliumromide) and colony formation, flow cytometric and transwell assays were performed to explore the cell function. Real-time PCR (RT-PCR) and Western blot were used to analyze the mRNA and protein expression. **Results:** In the present study, we found that C1QTNF6 significantly promoted the proliferation of SPCA1 and A549 cells by MTT and colony formation assays. In addition, down-regulation of C1QTNF6 weakened the tumor growth *in vivo*. Besides, C1QTNF6 remarkably reduced apoptosis by flow cytometric analysis and TUNEL assay. Furthermore, the capability of migration and invasion was obviously enhanced on C1QTNF6 overexpression. **Conclusion:** Overall, our results demonstrated that inhibition of C1QTNF6 attenuated cell proliferation, migration, invasion and promoted apoptosis *in vitro* and *in vivo* of NSCLC. Based on the above results, our study provided us with a new and key perspective in understanding and treating NSCLC.

## Introduction

Lung cancer is reported to be the most common malignant tumor in the world with a 5-year survival rate of <15% [1–3]. Lung cancer can be roughly divided into two types, small cell lung cancer (SCLC) and non-small cell lung cancer (NSCLC) by pathological classification [4,5]. Although great progress has been obtained about disease prevention, diagnosis and therapy improvement, the survival rate still remains at low level [6]. Therefore, the new effective markers for early stage diagnosis are the key to improve the cancer survival rates. Further investigations on the molecular mechanisms on NSCLC are essential for diagnosis and therapeutic strategies [5].

The C1q/tumor necrosis factor-related proteins (CTRPs) are a highly conserved family and have been found to be composed of 15 members (including CTRP1–CTRP15) [7]. C1qTNF6 [C1qTNF-related protein 6, also designated as CTRP6 (C1qTNF-related protein-6)] is a glycoprotein composed of 259 amino acids and has a unique molecular structure, consisting of four domains (signal peptide, short N-terminal variable region, collagen domain and C-terminal C1q domain) [8,9]. It expresses mainly in adipose tissue, lung, stomach and so on [10–12]. Growing body of evidences suggest that C1QTNF6 regulates cardiac fibrosis, inflammatory reaction, endothelial cell function, fibrogenesis, fatty acid metabolism and carcinogenesis and so on [13–17]. However, the function and detailed regulatory mechanism of C1QTNF6 in lung cancer remains unclear. The aim of the present study was to investigate the effect of C1QTNF6 on the progression of NSCLC.

## Materials and methods

### Tissues

Tumor tissues and paired adjacent normal tissues were obtained from 60 NSCLC patients who underwent surgery at Nanjing Medical University. All the tissue samples were collected and frozen in the liquid nitrogen, then stored at −80°C for future use.

### Cell culture and grouping

The NSCLC cell lines SPCA1 and A549 were supplied by American Tissue Culture Collection (ATCC; Rockville, U.S.A.) and cultured in RPMI-1640 medium supplemented with 10% Fetal Bovine Serum (FBS) in a 37°C, 5% CO_2_, and humidified incubator. The cells were divided into five groups: blank control group (Blank Control), si-control group (si-NC), si-C1QTNF6 transfection group (si-C1QTNF6) for down-regulated C1QTNF6, pcDNA3.1-control group (pc-NC), pcDNA3.1-C1QTNF6 transfection group (pc-C1QTNF6 group) for overexpressed C1QTNF6.

### RNA isolation and quantitative real-time PCR

Total RNA was extracted from cells using TRIzol reagent (Invitrogen, U.S.A.). Equal amounts of RNA were transcribed into cDNA using the cDNA First-Strand Synthesis kit (Life Technologies, U.S.A.). Total cDNA was used as a starting material for real-time PCR (RT-PCR) using the Step One Real-Time PCR System (Life Technologies Corp), and each sample was measured in triplicate. The PCR program was as follows: 95°C for 3 min followed by 40 cycles of 95°C for 10 s, and 60°C for 30 s. All fold changes were calculated using the 2^−ΔΔ*C*_T_^ comparative method using U6 for normalization.

### Western blot analysis

Each group of cells were lysed to extract total protein using RIPA lysis buffer. The protein concentrations were determined through Bicinchoninic Acid (BCA) Protein Assay Kit (Vazyme, U.S.A.). Equal amounts of protein (30 μg) were fractionated on a 10% sodium dodecyl sulfate (SDS) polyacrylamide gels, transferred to polyvinylidene difluoride (PVDF) membranes. The membranes were then blocked in 5% skim milk in Tris-buffered saline Tween (TBST) for 1.5 h. Subsequently, incubated with specific primary antibodies, including anti-P21 (bs55160R, Bioss, Beijing, China), anti-P27 (26714-1-AP, Proteintech, China), anti-CyclinD1 (bs20596R, Bioss, Beijing, China), anti-caspase-3 (bs0081R, Bioss, Beijing, China), anti-caspase-9 (bs0050R, Bioss, Beijing, China), anti-Bax (bs28034R, Bioss, Beijing, China), anti-Bcl-2 (bs34012R, Bioss, Beijing, China), anti-MMP-2 (10373-2-AP, Proteintech, China), anti-MMP-9 (10375-2-AP, roteintech, China), anti-GAPDH (10494-1-AP, Proteintech, China), at 4°C overnight. It was incubated with Horseradish Peroxidase–conjugated secondary antibody for 1 h at room temperature after washing with TBST. Protein expressions were detected using an Enhanced Chemiluminescence Detection System (Bio-Rad, CA, U.S.A.). GAPDH was used as a loading control.

### Cell proliferation assay

For 3-(4,5)-dimethylthiahiazo(-z-y1)-3,5-di-phenytetrazoliumromide (MTT) assay, transfected cells were seeded into 96-well plates and incubated for 24, 48, 72 h. MTT assay was applied to measure cell viability by detecting the solution absorbance at 550 nm wavelength with Microplate Reader (Thermo Fisher Scientific Inc., Waltham, MA, U.S.A.). For colony formation assay, cells were seeded in a six-well plate at a density of 1000 cells/well for 2 weeks, after which clones were fixed with methanol and stained with 0.1% Crystal Violet for 4 h at room temperature, and counted under a light microscope (Olympus, Tokyo, Japan).

### Flow cytometry for cell cycle and apoptosis

The effect of C1QTNF6 on cell cycle was detected by Cell Cycle Kit and Cell apoptosis was evaluated by flow cytometry analysis using FITC-Annexin V/propidium iodide (PI) Apoptosis Detection kit (BD Pharmingen, San Diego, CA, U.S.A.). After transfection for 24 h, SPCA1 and A549 cells were cultured for 24 h and washed twice with PBS. Then 20 μl of Annexin V and PI were added to each group and incubated for 15 min in the dark. The cell cycle and percentage of apoptotic cells was detected by FACS Calibur (BD Biosciences, U.S.A.). All analyses were performed in triplicate.

### Wound healing assay

Cells were seeded into six-well plate at 5 × 10^6^ cells/well and cultured at 37°C. When the cells reached confluence, a straight line scratched on the cell monolayer and width of the scratch was recorded under a microscope. Afterward, cells were washed twice with culture medium and cultured for 0, 24, 48 or 72 h. Cells were observed and photographed under a light microscope (Olympus, Tokyo, Japan).

### Transwell assay

Transwell assay was applied to measure cell migration and invasion ability of NSCLC cells. For migration assay, a total of 4 × 10^4^ cells/well in medium supplemented with 5% FBS were placed in the upper transwell chamber (Corning, Cambridge, MA), medium containing 20% FBS was added to the lower chambers and cultured at 37°C, 5% CO_2_ for 48 h. For invasion assay, cells were added to the upper transwell chambers, which were pre-coated with Matrigel. Other operations were the same as above. Then cells were fixed with 4% formaldehyde and stained with 0.1% Crystal Violet. The number of cells was counted under a microscope at 200× magnification.

### Tumor xenograft model in nude mice

All laboratory animals were cultivated and worked upon in Laboratory Animal Center, Nanjing Medical University. Fify microliters of 1 × 10^6^ SPCA1 and A549 cells which transfected si-NC, si-C1QTNF6, pc-NC or pc-C1QTNF6 were injected subcutaneously into the axilla of nude mice. The experimental mice were routinely monitored and killed on day 35 as per protocols set by the Ethical Committee of the Nanjing Medical University; the tumors were removed and tumor volume was measured as follows according to the formula: V (cm^3^) = width^2^ (cm^2^) × length (cm)/2. For Hematoxylin/Eosin (HE) staining, tumor tissues were fixed in 4% paraformaldehyde, and then embedded in paraffin and serially sectioned. The sections were stained with HE. Slides were visualized under a light microscope and at least five different sections of tumor tissues were detected for each group. All experimental animals were killed by inhaling carbon monoxide gas.

### Immunohistochemical analysis

Isolated tumor tissues from different groups were immunohistochemically stained for C1QTNF6. In brief, samples were fixed in 4% neutral formalin for 24 h and cut into 4-μm-thick sections, dried, deparaffinized and dehydrated in a graded ethanol series, and finally incubated with H_2_O_2_. Sections were incubated with primary antibodies at 4°C for 12 h. Then, secondary antibodies were applied to slides for 1 h at room temperature. The slides were incubated with 3,3′-diaminobenzidine (DAB) and counterstained with Hematoxylin. All the slides were visualized under a light microscope (Olympus, Tokyo, Japan).

### Statistical analysis

Statistical analysis was performed using SPSS 19.0 statistical software. Experimental data were expressed as mean ± SD. Differences between two groups were calculated by Student’s *t* test and multiple-group comparison were calculated by one-way analysis of variance (ANOVA) test followed by Tukey’s multiple comparison test of variance. *P*<0.05 were considered statistically significant.

## Results

### C1QTNF6 promoted NSCLC cell proliferation

**Figure 1:**
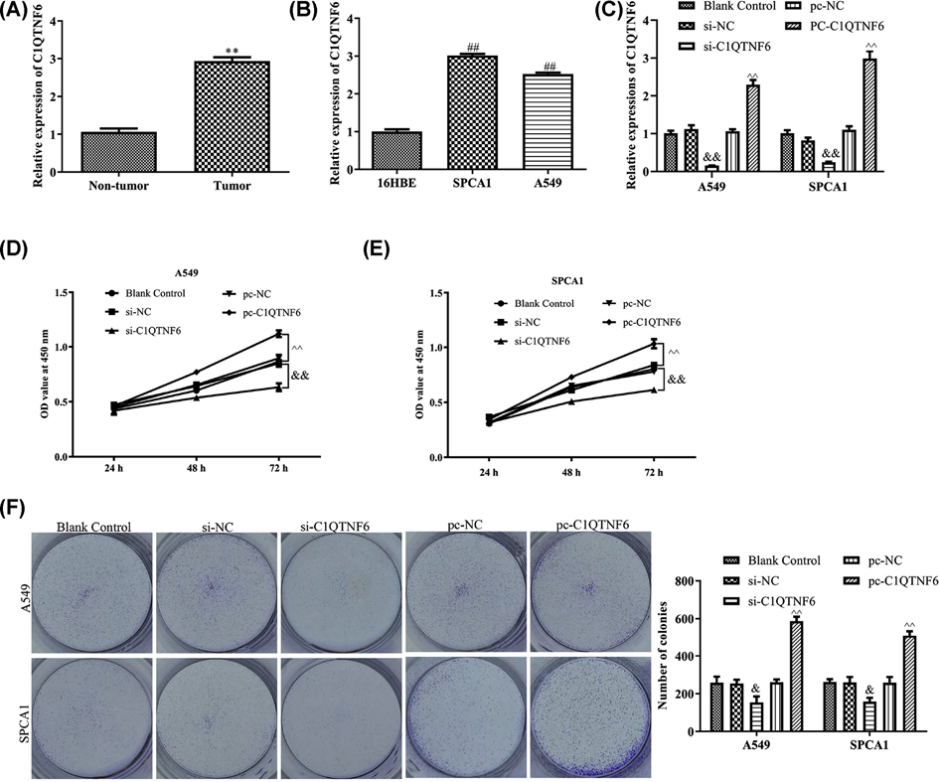
C1QTNF6 promoted NSCLC cell proliferation (**A**–**C**) RT-PCR showed expression level of C1QTNF6 in NSCLC tissues and cell lines. (**D,E**) MTT assay was performed to investigate the role of C1QTNF6 in viability of SPCA1 and A549 cells. (**F**) Colony formation assay was used to determine the effects of C1QTNF6 on colony formation abilities of SPCA1 and A549 cells. The results were expressed as the mean ± SD of three independent experiments and each was performed in triplicate. **P<0.01 vs. Non-tumor group, ^##^*P*<0.01 vs. 16HBE group, ^&&^*P*<0.01 vs. si-NC group, ^∧∧^*P*<0.01 vs. pc-NC group.

As shown in Figure 1A, RT-qPCR analysis revealed that the RNA expression level of C1QTNF6 was increased in the NSCLC tissues compared with that in the paired adjacent normal tissues. Consistent with the above results, the RNA expression level of C1QTNF6 was increased in two NSCLC cell lines (SPCA1 and A549) compared with the normal lung cell line (16HBE, Figure 1B).

To explore the role of C1QTNF6 in NSCLC cells, si-NC, si-C1QTNF6, pc-NC or pc-C1QTNF6 were transfected into SPCA1 and A549 cells separately. RT-qPCR assays was employed to measure the expression level of C1QTNF6 in NSCLC cells after infection, si-C1QTNF6 markedly down-regulated C1QTNF6 expression and pc-C1QTNF6 up-regulated C1QTNF6 expression compared with the controls (Figure 1C). Both MTT assay and colony formation assay were used to evaluate the impact of C1QTNF6 on proliferation of SPCA1 and A549 cells. MTT assay indicated that the viability of SPCA1 and A549 cells was enhanced in pc-C1QTNF6 group, which was contrast with si-C1QTNF6 group (Figure 1D,E). The same results were obtained in the colony formation experiment (Figure 1F). These data indicated that C1QTNF6 promoted NSCLC cell proliferation.

### Effect of C1QTNF6 on cell cycle in NSCLC

**Figure 2:**
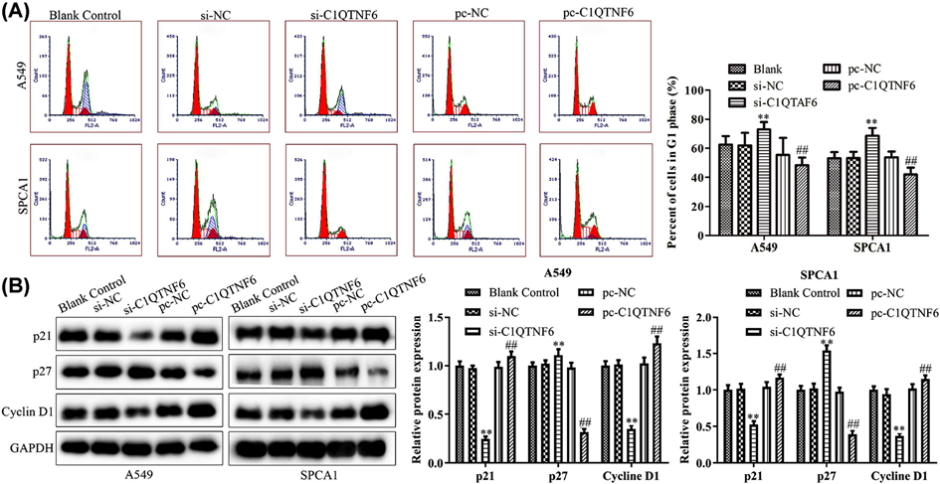
Effect of C1QTNF6 on cell cycle in NSCLC (**A**) Flow cytometry analysis was performed to evaluate the effects of C1QTNF6 on the cell cycle of SPCA1 and A549 cells. (**B**) The expression level of cycle-related proteins were explored in SPCA1 and A549 cell lines in different groups. **P<0.01 vs. si-NC group, ^##^*P*<0.01 vs. pc-NC group.

Flow cytometric analysis was adopted to test the activity of C1QTNF6 on cell cycle in NSCLC cells. As shown in Figure 2A, the cells transfected with si-C1QTNF6 had increased cell numbers in the G_1_ phase. Furthermore, up-regulated C1QTNF6 exhibited a reduction trend in the cell population in the G_1_ phase, indicating that knocking down C1QTNF6 could induce cell arrest in G_1_ phase in both SPCA1 and A549 cells. Besides, to verify this result, Western blotting assays were performed to detect the expressions of cell cycle-associated proteins. Knocking down of C1QTNF6 expression could down-regulate the levels of p21 and Cyclin D1, while C1QTNF6 overexpression induced the protein expressions of p21 and Cyclin D1 when compared with the blank control and si-NC groups (Figure 2B).

### C1QTNF6 inhibited NSCLC cell apoptosis

**Figure 3:**
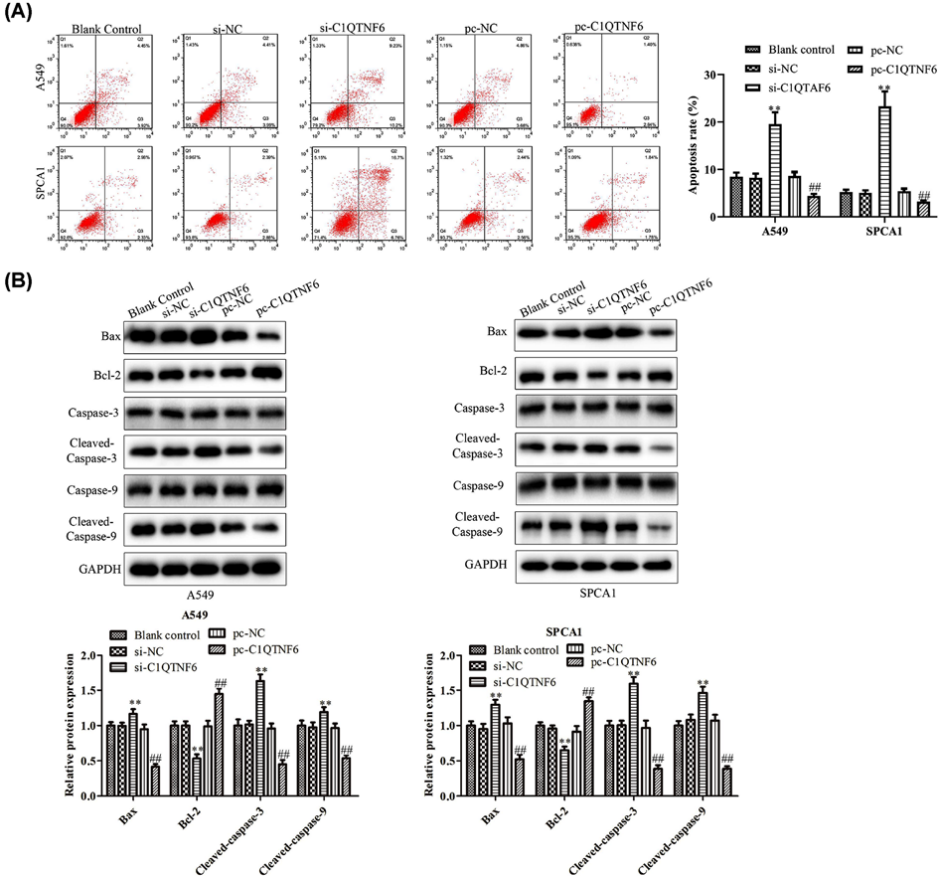
C1QTNF6 inhibited NSCLC cell apoptosis *in vitro* (**A**) Flow cytometry analysis was performed to evaluate the effects of C1QTNF6 on the apoptosis of SPCA1 and A549 cells. (**B**) The expression level of apoptosis-related proteins caspase-3, caspase-9, Bcl-2 and BAX in SPCA1 and A549 cell lines in different groups. **P<0.01 vs. si-NC group, ^##^*P*< 0.01 vs. pc-NC group.

Flow cytometry analysis was used to determine whether C1QTNF6 could aggravate apoptosis of SPCA1 and A549 cells. As expected, the results of cell apoptosis have shown that overexpression of C1QTNF6 remarkably suppressed apoptosis of SPCA1 and A549 cells. Conversely, down-regulation of C1QTNF6 induced an increase in the proportion of apoptotic cells of SPCA1 and A549 cells compared with control group (Figure 3A). Further, the expression levels of apoptosis-related proteins were determined by Western blot analysis. Compared with the controls, the expression levels of Bax, cleaved-caspase-3, and cleaved-caspase-9 were down-regulated when C1QTNF6 up-regulated. Furthermore, depletion the C1QTNF6 expression caused opposite results (Figure 3B).

### C1QTNF6 promoted the migration and invasion of NSCLC cells

**Figure 4:**
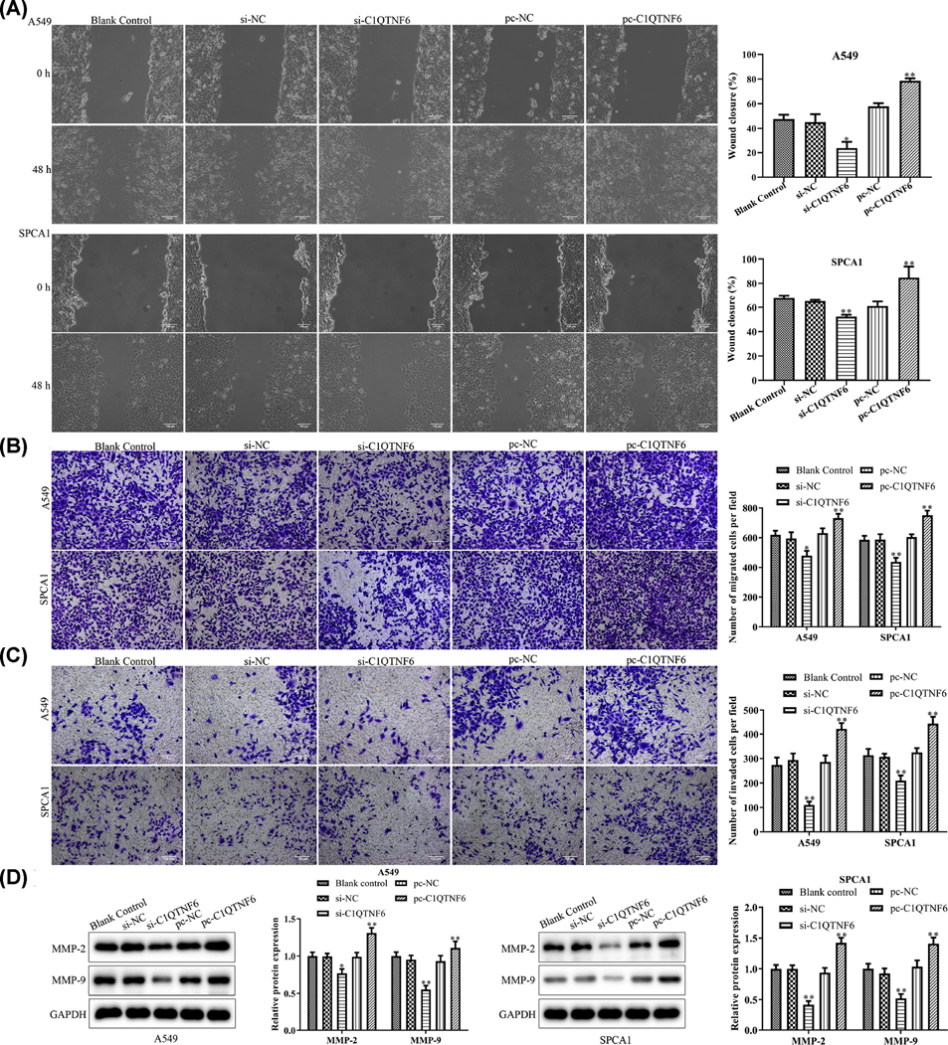
C1QTNF6 promoted the migration and invasion of NSCLC cells (**A**) Wound healing assay and (**B**) Transwell migration analysis were performed to examine the effects of C1QTNF6 on migration abilities of SPCA1 and A549 cells. (**C**) Transwell invasion assay was carried out to examine the role of C1QTNF6 in invasion ability of SPCA1 and A549 cells. (**D**) Western blotting assay was used to assess the effects of liquiritin on the expressions of MMP-2 and MMP-9. The band intensity was quantified by ImageJ software. The results were expressed as the mean ± SD of three independent experiments and each was performed in triplicate. *P <0.05 vs. si-NC group, **P<0.01 vs. si-NC group, ^##^*P*<0.01 vs. pc-NC group.

To further elucidate how C1QTNF6 influences cell migration and invasion abilities, wound-healing, transwell migration and invasion assays were applied. As shown in Figure 4A, the wound healing rates in si-C1QTNF6 groups were significantly depressed compared with that of control group. Furthermore, transwell assay demonstrated that down-regulation of C1QTNF6 inhibited the migration ability of SPCA1 and A549 cells (Figure 4B). Overexpression of the C1QTNF6 caused opposite results. As shown in Figure 4C, the cells that transfected with the pc-C1QTNF6 were distinctively more invitatory than control cell. Meanwhile, Western blotting was adapted to detect the MMP-2 and MMP-9 levels. We found that the levels of MMP-2 and MMP-9 dramatically increased in pc-C1QTNF6 group, while decreased compared with control group (Figure 4D). Overall, these results revealed that a positive effect of C1QTNF6 in migration and invasion ability of SPCA1 and A549 cells.

### C1QTNF6 promoted the development of NSCLC xenografts *in vivo*

**Figure 5:**
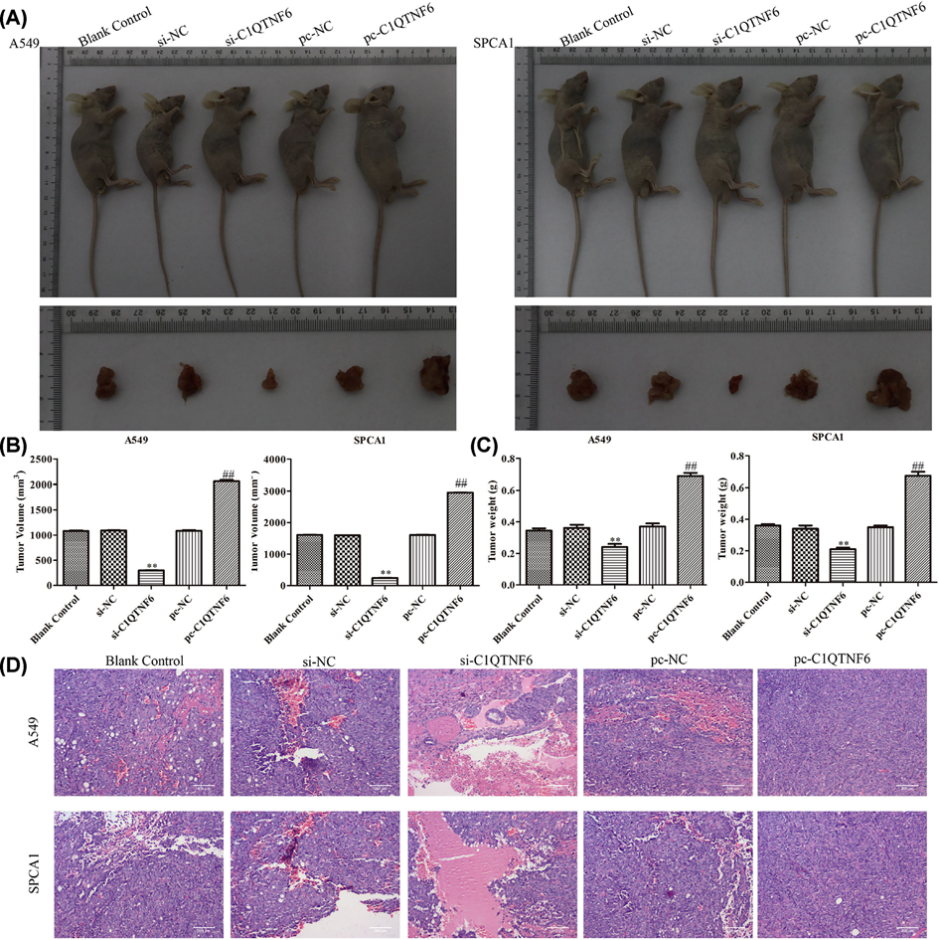
C1QTNF6 promoted the development of NSCLC xenografts *in vivo* (**A**) Mice and tumors in the different groups are shown. (**B**) Tumor volume, (**C**) tumor weight are recorded. (**D**) HE assay is presented. All data are shown as mean ± SD (*n*=6). **P<0.01 vs. si-NC group, ^##^*P*<0.01 vs. pc-NC group.

The effects of C1QTNF6 were further confirmed on NSCLC xenografts *in vivo*. SPCA1 and A549 cells transfected with blank, control siRNA, si-C1QTNF6 and pc-C1QTNF6 were injected into the axilla of the male Balb/c nude mice. After the experimental nude mice were killed, the tumors were weighted and volumes were measured. The results of Figure 5A showed that si-C1QTNF6 could significantly suppress the xenograft tumors growth comparing with the control group. Furthermore, tumor volumes and weights were also significantly decreased in si-C1QTNF6 group (Figure 5B,C). However, up-regulation of C1QTNF6 promoted the tumor growth and weight. Additionally, the results of HE assay indicated that nude mice from pc-C1QTNF6 group had more severe damage in tumor tissues compared with those in control group (Figure 5D).

### The effect of C1QTNF6 on the invasion and apoptosis of NSCLC *in vivo*

**Figure 6:**
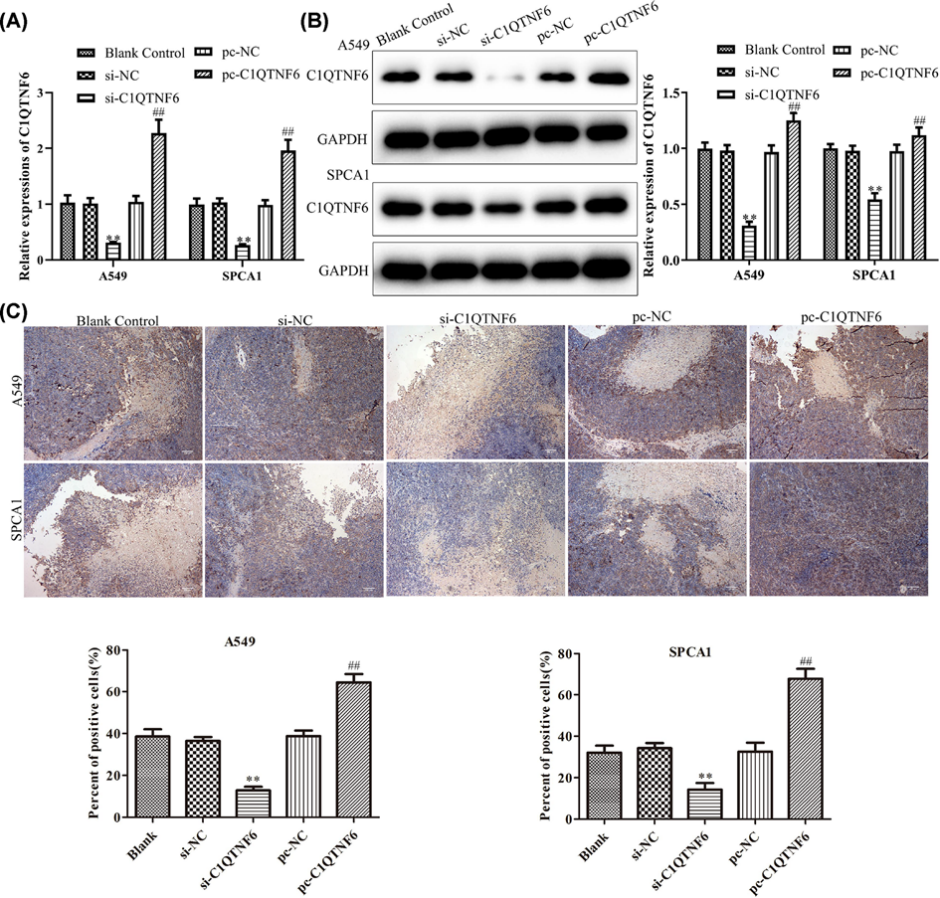
Expression of C1QTNF6 in different groups (**A**–**C**) RT-PCR, Western blotting and immunohistochemistry analyses were adapted to evaluate the expressions of C1QTNF6 in tumors. **P<0.01 vs. si-NC group, ^##^*P*<0.01 vs. pc-NC group.

**Figure 7:**
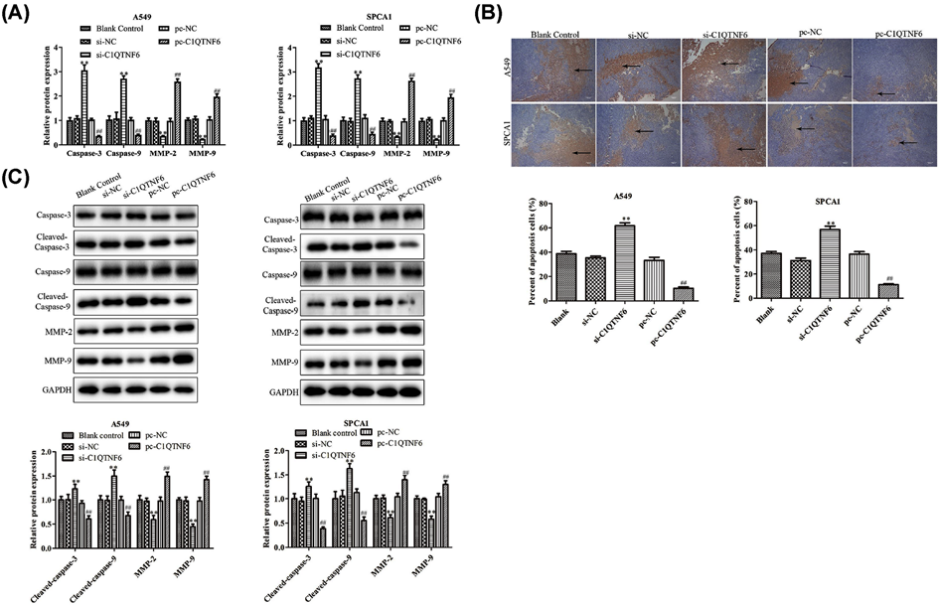
C1QTNF6 inhibited NSCLC cell apoptosis *in vivo* (**A**) RT-PCR and Western blotting assay was used to assess the effects of C1QTNF6 on the expressions of MMP-2 and MMP-9. (**B**) TUNEL assay was performed to evaluate the effects of C1QTNF6 on the apoptosis of tumors. (**C**) The expression level of apoptosis-related proteins caspase-3, caspase-9, Bcl-2 and BAX in different groups. **P<0.01 vs. si-NC group, ^##^*P*<0.01 vs. pc-NC group.

RT-PCR, Western blotting and immunohistochemistry analysis were adapted to evaluate the expressions of C1QTNF6 in tumors. As shown in Figure 6A–C, the expression of C1QTNF6 was significantly suppressed in si-C1QTNF6 group, while obviously increased in pc-C1QTNF6 group compared with control group. At the same time, we examined the expression levels of migration-related proteins using RT-PCR and Western blotting analysis. We found that knocking down C1QTNF6 obviously down-regulated MMP-2 and MMP-9 expressions. In addition, the expression of MMP-2 and MMP-9 were increased in pc-C1QTNF6 group (Figure 7A). As shown in Figure 7B, the arrow represents TUNEL-positive cells, the results suggested that down-regulated C1QTNF6 expression could significantly promote tumor cell apoptosis, while C1QTNF6 overexpressed inhibited the tumor cell apoptosis *in vivo* relative to the control group. Besides, we observed an increase in the protein expressions of cleaved-caspase-3, cleaved-caspase-9, MMP-2 and MMP-9 in si-C1QTNF6 group, which was in contrast with pc-C1QTNF6 group compared with control group (Figure 7C). These data indicated that si-C1QTNF6 exhibited anti-cancer activity *in vivo*.

## Discussion

In the present study, we found C1QTNF6 was significantly highly expressed in NSCLC tissues and cells. For the first time, we provided direct evidences that C1QTNF6 acted as an oncogene and promoted NSCLC cell growth and metastasis.

Lung cancer is reported to be the most common malignancy and leading cause of cancer-related deaths in terms of both morbidity and mortality worldwide [18–21]. At present, the most effective treatment for NSCLC is obtained, but it is easy to recur and has poor prognosis [22], the overall 5-year survival rate has not improved significantly [23,24]. Given these data, it emphasizes an urgent need for new therapeutic targets with high efficiency for NSCLC diagnosis and treatment. Therefore, more and more studies have focused on exploring new and effective markers for early diagnosis of recurrence and metastasis, providing potential therapeutic targets for NSCLC [25,26].

Presently, accumulating evidence demonstrated that C1QTNF6 have important roles in human disease progression and metastasis, including cardiac fibrosis, inflammatory reaction, endothelial cell function, fibrogenesis, fatty acid metabolism and carcinogenesis, and so on [13–17]. For instance, Chi et al. have reported that C1QTNF6 improves PPARγ activation to alleviate angiotensin II-induced hypertension and vascular endothelial dysfunction in spontaneously hypertensive rats; Murayama et al. demonstrated that C1QTNF6 is an endogenous complement regulator that can effectively treat induced arthritis; and in 2019, Han et al. revealed that C1QTNF6 as a novel biomarker regulates cellular behaviors in A549 cells and exacerbates the outcome of lung adenocarcinoma patients. However, the expression of C1QTNF6 in NSCLC was still unknown and its function and detailed regulatory mechanism merited further investigation. In the present study, the experiments suggested that ectopic expression of C1QTNF6 significantly promoted cell proliferation of SPCA1 and A549 cells and tumor growth *in vivo*.

Apoptosis is strictly regulated by many proteins and pathways. Bcl-2 family members play an important role in regulating apoptosis [27]. The Bcl-2 family consists of pro-apoptotic molecules (Bax, Bim, Bcl-xs, Bak, Bid, Bad, Bik) and anti-apoptotic (Bcl-2, Bcl-xl, Bcl-w, A1) [28]. Bax proteins allow small molecules such as ions and cytochrome *c* to penetrate the mitochondrial membrane into the cytoplasm which leads to cell apoptosis. In our study, compared with the controls, the levels of Bax, caspase-3, and caspase-9 were down-regulated on C1QTNF6 overexpression. However, our results detected the decreased Bcl-2 expression in SPCA1 and A549 cells with C1QTNF6 down-expression, while the C1QTNF6 overexpression showed opposite results in the cells. Additionally, knocking down C1QTNF6 obviously induced the apoptosis of SPCA1 and A549 cells. On the contrary, overexpression of C1QTNF6 showed a decrease in apoptosis in both cell lines. Moreover, down-regulation of C1QTNF6 could weaken the tumor growth and apoptosis *in vivo*. These findings suggested that C1QTNF6 might act as a tumor oncogene and down-regulation of its expression may contribute to the progression of NSCLC.

Tumor spreading of cancer to bones, lungs and brain largely depends on the ability of tumor cells to invade the adjacent tissues, which also successfully establishes a metastatic tumor. Tumor metastasis is a complex, efficient and lethal event, and the main cause of death in cancer patients [29]. Therefore, prevention of cancer cell metastasis is an effective strategy for successful management of cancers. At the same time, *in vivo* and *in vitro* experiments also demonstrated that C1QTNF6 could substantially promote the ability of migration and invasion of NSCLC cells by wound healing and transwell assays.

In conclusion, our results suggested that that inhibition of C1QTNF6 attenuated cell proliferation, migration, invasion and promoted apoptosis *in vitro* and *in vivo* of NSCLC. It provides us with a new and key perspective in understanding and treating NSCLC.

## Data Availability

All data generated or analyzed during the present study are included in this published article.

## Abbreviations

CTRP: C1q/tumor necrosis factor-related protein
C1QTNF6: C1q/tumor necrosis factor-related protein 6
FBS: fetal bovine serum
GAPDH: glyceraldehyde-3-phosphate dehydrogenase
HE: Hematoxylin/Eosin
MMP: matrix metalloprotein
MTT: 3-(4,5)-dimethylthiahiazo(-z-y1)-3,5-di-phenytetrazoliumromide
NSCLC: non-small cell lung cancer
PI: propidium iodide
PPAR: peroxisome proliferators-activated receptor
RIPA: Radio Immunoprecipitation Assay
RT-PCR: real-time PCR
TBST: Tris-buffered saline Tween
TUNEL: TdT-mediated dUTP Nick-End Labeling
U6: U6 small nuclear RNA
