# Expression of Concern: C1QTNF6 regulates cell proliferation and apoptosis of NSCLC in vitro and in vivo

**DOI:** 10.1042/BSR-2020-1541_EOC

**Published:** 2024-10-29

**Keywords:** apoptosis, C1QTNF6, NSCLC, proliferation

The Editorial Office has been made aware of potential issues surrounding the scientific validity of this paper “C1QTNF6 regulates cell proliferation and apoptosis of NSCLC in vitro and in vivo” DOI: 10.1042/BSR20201541, hence has issued an expression of concern to notify readers. It has been noted that Figure 4C A549 si-C1QTNF6 panel in this paper seems to appear as Figure 7E A549/sh-SNHG3 Invasion NC inhibitors panel in a paper with overlapping submission dates: Zhao et al, 2021 (DOI: 10.3892/ijmm.2021.4980) [retracted]. Additionally, Figure 1F A549 Blank Control, A549 si-NC, and SPCA-1 pc-C1QTNF6 panels seem to appear as Figure 2A Solvent control, LPS+ATP, and LPS+ATP+50µM Lovastatin panels in a subsequent publication from Liu et al, 2022 (DOI: 10.21037/tcr-22-346) [retracted].

The authors have been contacted regarding these concerns and have corresponded with the journal. The authors state the results are their own work and provided the original data pertaining to their article, for which no further issues have been identified. However the authors' response did not fully resolve the concerns regarding the overlapping data with the other articles.

